# Study on Joint Model Simplification for Finite Element Analysis of Bamboo/Wood-Oriented Strand Board Furniture

**DOI:** 10.3390/ma17174395

**Published:** 2024-09-06

**Authors:** Kaiting Zhang, Jun Zhang, Yong Guo, Yuxia Chen

**Affiliations:** 1 School of Fine Arts and Design, Chaohu University, Hefei 238024, China; 2 School of Machinery Engineering, Chaohu University, Hefei 238024, China; 13705699844@163.com; 3 College of Forest and Garden, Anhui Agricultural University, Hefei 230036, China; guoyong@ahau.edu.cn

**Keywords:** model simplification, joint elastic modulus, bamboo board, oriented strand board, finite element analysis, L-shaped component

## Abstract

Board furniture’s performance and scientific design are making it popular. Research on simplifying furniture joints reduces design cycles and costs and improves structural safety. In this article, using a cantilever beam to calculate deflection theoretically simplifies the L-shaped component model and yields a joint elastic modulus formula. Finite element analysis (FEA) confirms the effectiveness of this simplified model by comparing its results with experimental data. In simplified components, the joint elastic modulus increases with length (*l*_2_) and stabilizes at *l*_2_/*b* ≥ 6 (*b* is the board’s thickness). The variation pattern of the joint elastic modulus equals that of the stiffness, proving its usefulness in assessing component deformation resistance. Furthermore, the component strength and stiffness are also affected by the screw spacing and connector type. In particular, the connectors type affects bamboo-oriented strand board (BOSB) component performance more than wood-oriented strand board (WOSB). Compared to WOSB, BOSB components have superior strength and stiffness and are more stable. The recommended screw spacing for L-shaped components is 48 mm. BOSB components fixed with two-in-one and metal nuts utilizing threads embedded in the board have better strength and stiffness, while for WOSB components, nylon nuts, and wooden dowel pins are more appropriate for securing.

## 1. Introduction

As home consumption demand continues to rise, the board furniture sector has increasingly become a significant component of the furniture industry [1]. Currently, the national board furniture industry is in a period of expansion. According to statistics, the production of board furniture has surpassed 70% of the total output of the entire furniture business. There are over 500 domestic furniture brands, with board furniture making up approximately one-third of the total. Consumers highly regard board furniture for its steady material performance, its ability to be disassembled and reassembled as desired, its cost-effectiveness, its diverse designs, and its fashionable appearance. China is one of the countries with the richest bamboo resources, known as the “bamboo kingdom” [2]. Moreover, bamboo utilization in terms of production varieties, scale, and output is among the highest in the world [3,4,5]. At present, the production and exploitation of boards account for the main aspect of bamboo utilization, which is widely used in furniture, interior decoration, construction, packaging, etc., making an important contribution to the economic development of China [6,7]. Among them, bamboo-oriented strand board (BOSB) using large bamboo flakes not only improves the utilization of bamboo [8,9], but the board also has the advantages of excellent mechanical properties [10] and good dimensional stability [11,12], making it potentially valuable as board furniture material. Board furniture refers to the utilization of artificial boards and hardware to construct furnishings, with the weakest point often being the connection between the boards [13,14]. Many studies have used the robustness of corner components to assess the mechanical characteristics of the entire furniture. The majority of these researchers employ finite element analysis (FEA) to examine the structural integrity of different corner joints [15,16,17,18]. When compared to solid wood components, board materials offer more uniformity, a more consistent shape, easier unit discretization, and enhanced convenience for FEA [14,19].

When performing FEA, establishing proper contact between the connectors and boards, as well as between the boards themselves, is a crucial factor. Certain scholars classify this interaction as bonded, while others advocate for characterizing it as frictional contact; however, accurately determining the friction coefficient proves challenging [20,21,22]. Furthermore, to precisely replicate the forces exerted on the components, some studies have incorporated intricate characteristics (such as threads) into their models. Nevertheless, issues with contact settings and mesh division precision during FEA can result in incorrect outcomes and affect the accuracy and effectiveness [19,23]. Some scholars have simplified the FEA components. For instance, Nicholls et al. employed elastic finite element components to substitute joints and considered joints as a unified entity while investigating the strength of joints in standard box-type structures [24]. Similarly, Krzyzaniak et al. utilized a simplified model that incorporated predetermined friction coefficients to investigate the response of impact damage in L-shaped joints [13]. Koc et al. used SolidWorks and CosmosWorks software to streamline the connection fixation of board components while conducting shelf deflection analysis [25]. These studies illustrate that simplified models can enhance the efficiency of FEA while producing comparable results to experimental data. Therefore, this paper regards the simplification of components in board furniture as a crucial research focus that directly influences the computational expense of FEA [26].

The aforementioned studies employed a simplification or omission of the connector model features [2]. However, in FEA, it is still necessary to take into account the contact between the board and the connectors, as well as between the boards [24,27,28]. This consideration continues to impact the analysis process and efficiency. Smardzewski et al. introduced a technique for determining the elastic modulus of joints, in which the corner joint is treated as an essential component and simplified [29]. The component model directly assigned the elastic modulus, eliminating the need to model the connectors. However, the paper solely focused on the collective contact area of the two boards and connectors without investigating the impact of the simplified area size on the simulation outcomes.

Accordingly, this study aims to assess the strength of L-type components made of oriented strand board using physical tests and theoretical derivation. Additionally, it examines the impact of model simplification on the joint elastic modulus. Furthermore, FEA is employed to evaluate the accuracy of various simplified models. This research provides a method for predicting and optimizing the structural strength of board furniture, making its design more scientific and cost-effective for furniture enterprises.

## 2. Materials and Methods

### 2.1. Properties of Selected Materials

**Figure 1:**
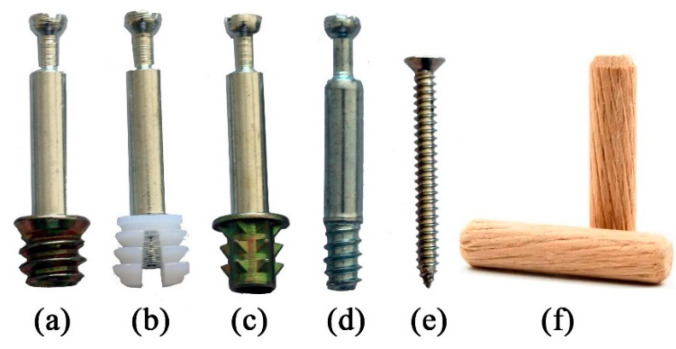
L-type connectors: (**a**) metal nut; (**b**) nylon nut; (**c**) converse-spine nut; (**d**) two-in-one connector; (**e**) screw; and (**f**) wooden dowel pin.

For this experiment, we selected two types of boards, BOSB (Anhui Longhua Bamboo Flooring Co., Ltd, Lu’an, China) and wood-oriented strand board (WOSB) (Shanghai Huqiang Wood Industry (Group) Co., Ltd., Shanghai, China), which are considered to be isotropic parallel to their wide surfaces. The adhesive used in both boards was PMDI (polymeric methylene di isocyanate). A previous article thoroughly explains the procedure for testing the material properties, and Table 1 displays the results [19]. Table 2 and Figure 1 display the various types, materials, and dimensions of the connectors.

### 2.2. Preparation of Joints

**Figure 2:**
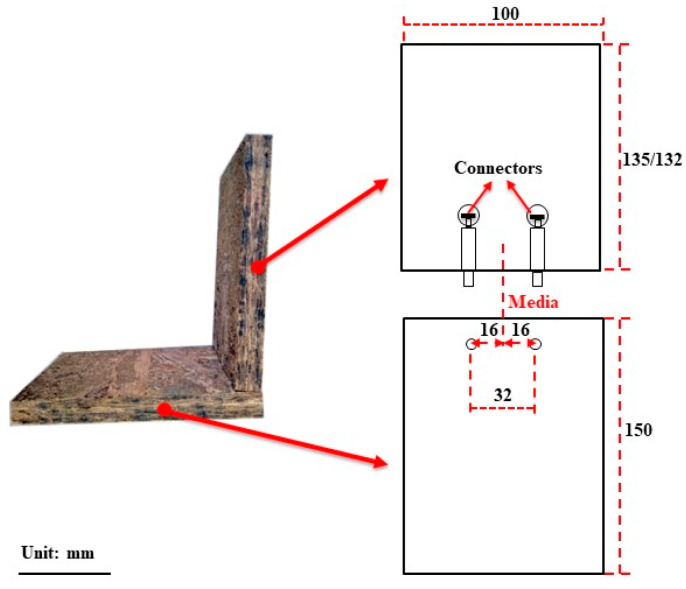
Dimensions of L-type component.

Self-drilling screws were bought from a local commercial supplier (Hefei, China). The dimensions of the BOSB were 150 × 100 × 15 mm and 135 × 100 × 15 mm, and the dimensions of the WOSB were 150 × 100 × 18 mm and 132 × 100 × 18 mm. The screw spacings were 16, 32, 48, 64, and 80 mm. The spacings for the eccentric connectors and dowels were 32 mm. As shown in Figure 2, the connectors were evenly distributed and secured at the centerline in the direction of the board thickness. Table 3 illustrates the determination of the aperture ratio based on the previous test period results, leading to the selection of the optimal perimeter ratio. Each component was prepared from 10 repetition samples, for a total of 200.

### 2.3. Stiffness Testing

**Figure 3:**
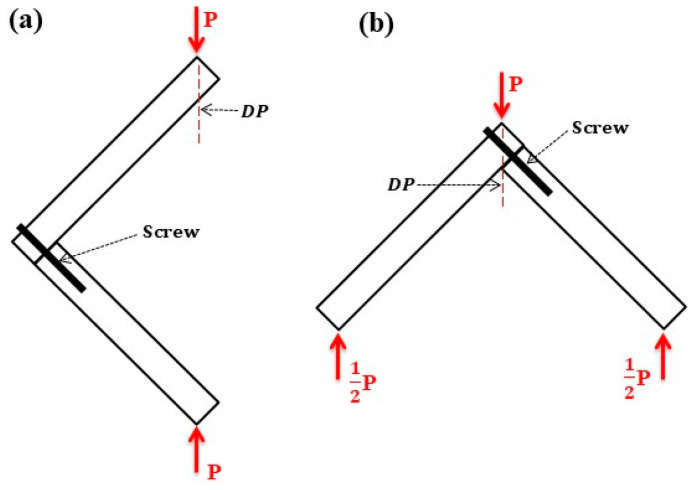
A diagram of loads during (**a**) compression and (**b**) tension.

L-type components were loaded with external forces P according to the diagram presented in Figure 3. The tests were performed by a mechanical testing machine (model WDW-100E, Jinan Chenda Testing Machine Manufacture Co., Ltd., Jinan, China) in laboratory conditions (temperature of 21 ± 1 °C; air relative humidity of 65 ± 3%). The crosshead speed was 10 mm/min. The pressure head was cylindrical with a diameter of 150 mm. The experimental tests directly provided dependence between the force *P* and displacement *DP*. Several articles provide detailed descriptions of the methods for measuring the force and displacement of the component [20,30,31]. The stiffness of the components in tension and compression, *S_T_* and *S_C_* (N/m), was calculated as follows:

(1) $$S_{C}=\frac{0.4P_{max}-0.1P_{max}}{{DP}_{0.4Pmax}-{DP}_{0.1Pmax}}$$

(2) $$S_{T}=\frac{0.4\times 0.5P_{max}-0.1\times 0.5P_{max}}{{DP}_{0.4Pmax}-{DP}_{0.1Pmax}}$$

where *P_max_* is the maximum of the force (N); *DP_Pmax_* is the displacement at which the force reaches its maximum value (m); *DP*_0.4*Pmax*_ and *DP*_0.4*Pmax*_ are the displacements at which the force reaches 0.4 times and 0.1 times its maximum value, respectively.

### 2.4. Numerical Model of Joints

**Figure 4:**
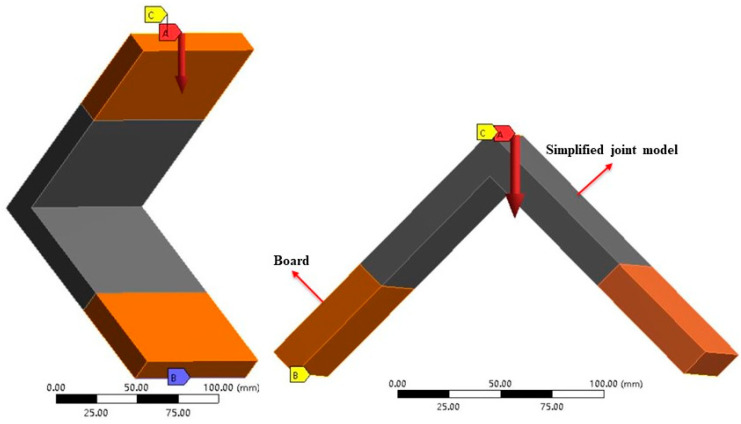
Numerical model of the experiment. A–C denote the applied boundary conditions.

Modeling and numerical simulations were performed using the Siemens NX 12.0 program and ANSYS Workbench 17.0 software, respectively. The model’s geometry, loading, and boundary conditions were based on those in Figure 4. The Young’s Modulus values of simplified joint models in the FEA were their joint elastic modulus, while the other parts were the Young’s Modulus of corresponding borads.

## 3. Results and Discussion

### 3.1. Derivation of the Joint Elastic Modulus Formula

The elastic modulus of the joint is a significant indicator of the component’s strength, with higher values indicating greater resistance to deformation [29]. Under small deformation conditions, the bending of the components’ arms can be considered equivalent to that of a combined cantilever beam. The deflection of the cantilever beam was calculated using the superposition technique. The theoretical calculation formula for component deformation established the correlation between the elastic modulus and the length of the simplified joint. Therefore, by adjusting the length of the joint and determining its elastic modulus, this value was used in the FEA of a simplified joint model to calculate its deflection. Subsequently, the deflection was compared with empirical data in order to determine the most favorable simplified model. The procedure for determining the elastic modulus when subjected to compressive and tensile loads for joints was as follows.

**Figure 5:**
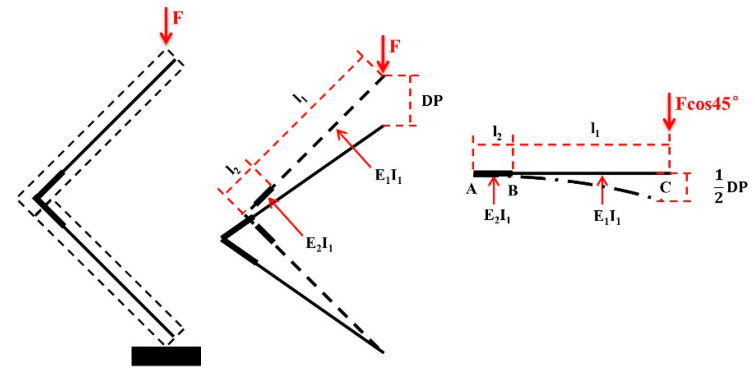
Force analysis of compression of component.

Figure 5 depicts the component simplification process during compression. The AB segment first underwent rigidifying, followed by analysis of the cantilever beam’s bending deformation.

(3) $$y_{c1}=-\frac{P{l_{1}}^{3}}{{3E_{1}I}_{1}}$$

When rigidifying the BC section, point B was subject to force *F* and torque *FI*, and the point’s deflection *y_B_* and angle *θ_B_* were calculated as follows:

(4) $$y_{B}=y_{BF}+y_{BM},\;\theta_{B}=\theta_{BF}+\theta_{BM}$$

(5) $$y_{BF}=-\frac{Pl_{2}^{3}}{{3E_{2}I}_{1}},\;y_{BM}=-\frac{Pl_{1}l_{2}^{2}}{{2E_{2}I}_{1}}$$

(6) $$\theta_{BF}=-\frac{Pl_{2}^{2}}{{2E_{2}I}_{1}},\;\theta_{BM}=-\frac{Pl_{1}l_{2}}{{E_{2}I}_{1}}$$

The deflection at point C, denoted as *y*_*C*2_, is influenced by the deformation at point B and equal to the sum of the deflection at point B and the upward deflection (*θ*_*B*_*l*_1_) at point C caused by the angle of rotation *θ_B_*.

(7) $$y_{C2}=y_{B}+\theta_{B}l_{1}$$

The deflection at point C is as follows:

(8) $$y_{C}=y_{C1}+y_{C2}$$

The following is obtained:

(9) $$y_{C}=\frac{Pl_{1}^{3}}{3E_{1}I_{1}}+\frac{Pl_{2}^{3}+3Pl_{1}l_{2}^{2}+3Pl_{2}l_{1}^{2}}{3E_{2}I_{1}}$$

The deflection at point C is obtained:

(10) $$DP={2y}_{C}cos\alpha$$

Solving this equation, the following is obtained:

(11) $$E_{2}=\frac{FE_{1}(l_{2}^{3}+3l_{1}l_{2}^{2}+3l_{2}l_{1}^{2})}{3{E_{1}I}_{1}DP-Fl_{1}^{3}}$$

where *P* is the vertical part of the force acting on the cantilever and has the value *F*cosα (α = 45°); F is the force collected during the experiment; *F* = 0.4*F*_max_ − 0.1*F*_max_; *F_max_* is the maximum of the force (N); *E*_1_ is the board’s elastic modulus; *E*_2_ is the joint’s elastic modulus; *I*_1_ is the board’s inertia moment; *I*_1_ = *Lb*^3^/12 (*b* is the board’s thickness (m) and *L* is its width (m)); *l*_1_ is the joint’s arms’ length (m); *l*_2_ is the simplified joint model’s length (m); *l*_2_ = *xb*, *x* ranges from 1 to 10; *DP* = *DP*_0.4*max*_ − *DP*_0.1*max*_; *DP_Pmax_* is the displacement at which the force reaches its maximum value (m).

**Figure 6:**
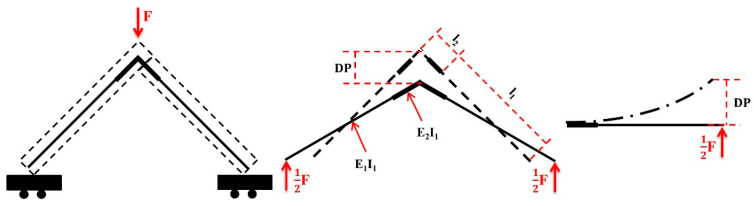
Force analysis of tension of component.

Under tension, the component’s force situation was similar to that under compression. However, the joint deflection under tension aligned with the cantilever deflection and did not display a 2:1 relationship. *P* = 1/2*F*cos*α* (*α* = 45°). Figure 6 shows the component simplification process in tension.

The following formula determines the deflection at point C of the tempered AB section of the cantilever beam:

(12) $$y_{c1}=-\frac{Pl^{3}}{{3E_{1}I}_{1}}$$

After tempering the BC section, the deflection *y_B_* and angle of turn B at point B were calculated as follows:

(13) $$y_{B}=y_{BF}+y_{BM},\;\theta_{B}=\theta_{BF}+\theta_{BM}$$

(14) $$y_{BF}=-\frac{Pl_{2}^{3}}{{3E_{2}I}_{1}},\;y_{BM}=-\frac{Pl_{1}l_{2}^{2}}{{2E_{2}I}_{1}}$$

(15) $$\theta_{BF}=-\frac{Pl_{2}^{2}}{{2E_{2}I}_{1}},\;\theta_{BM}=-\frac{Pl_{1}l_{2}}{{E_{2}I}_{1}}$$

The formula for the deflection at point C is as follows:

(16) $$y_{C2}=y_{B}+\theta_{B}l_{1}$$

(17) $$y_{C}=y_{C1}+y_{C2}$$

(18) $$y_{C}=\frac{Pl_{1}^{3}}{3E_{1}I_{1}}+\frac{Pl_{2}^{3}+3Pl_{1}l_{2}^{2}+3Pl_{2}l_{1}^{2}}{3E_{2}I_{1}}$$

(19) $$DP=y_{C}cos\alpha$$

As a result, the joint’s elasticity modulus under tension assumes the following form:

(20) $$E_{2}=\frac{PE_{1}(l_{2}^{3}+3l_{1}l_{2}^{2}+3l_{2}l_{1}^{2})}{{12E_{1}I}_{1}DP-Pl_{1}^{3}}$$

where *P* is the vertical part of the force acting on the cantilever and has the value *F*cosα (α = 45°); *F* is the force collected during the experiment; *F* = 0.4*F_max_* − 0.1*F_max_*; *F_max_* is the maximum of the force (N); *E*_1_ is the board’s elastic modulus; *E*_2_ is the joint’s elastic modulus; *I*_1_ is the board’s inertia moment; *I*_1_ = *Lb*^3^/12 (*b* is the board’s thickness (m) and *L* is its width (m)); *l*_1_ is the joint’s arms’ length (m); *l*_2_ is the simplified joint model’s length (m); *l*_2_ = *xb*, *x* ranges from 1 to 10; *DP* = *DP*_0.4*max*_ − *DP*_0.1*max*_; *DP_Pmax_* is the displacement at which the force reaches its maximum value (m).

### 3.2. Elastic Modulus of Joints

**Figure 7:**
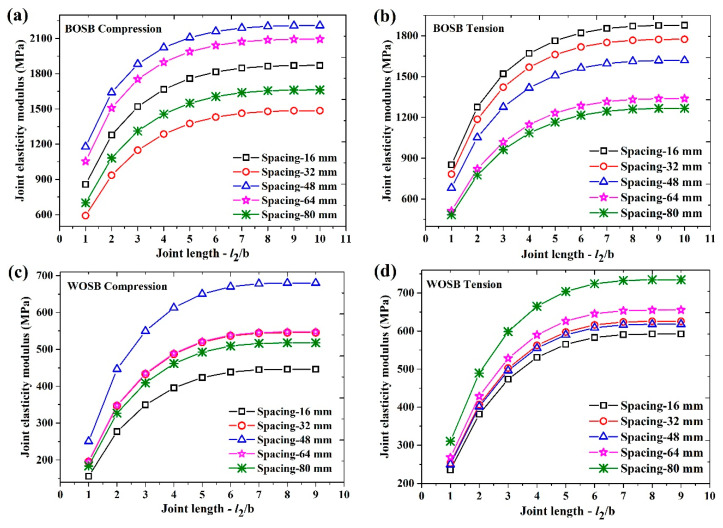
The joint elastic modulus of components fixed with screws: (**a**) BOSB—compression; (**b**) BOSB—tension; (**c**) WOSB—compression; (**d**) WOSB—tension.

Equations (11) and (20) calculate the joint elastic modulus of the L-shaped component. Figure 7 shows the joint elastic moduli of the BOSB and WOSB components. Figure 7a,b show that the joint elastic modulus increased proportionally to the length of the simplified BOSB joints. When the length of the simplified BOSB joints was six times the board’s thickness, the elastic modulus reached a stable state. Furthermore, the elastic modulus increased initially and then decreased as the spacing increased under compressive load. The BOSB component with a spacing of 48 mm had the highest elastic modulus, followed by those with spacings of 64 mm and 16 mm. The BOSB component with a spacing of 32 mm exhibited the lowest elastic modulus. Under tension, the elastic modulus decreased as the screw spacing increased. Similarly, in the case of the WOSB components, the joint elastic modulus initially increased and then decreased as the spacing increased. Furthermore, it was observed that the WOSB components with a 48 mm screw spacing had the highest compressive joint elastic modulus. This was the same for the BOSB components. As the screw spacing increased, the tension elastic modulus increased. Additionally, the BOSB components had a significantly higher joint elasticity modulus than the WOSB components. This is due to the strong dependence between the load of the connections and the density of the boards [32]. The mean density of the BOSB was 806.61 kg·m^−3^, which is about 1.4 times that of the WOSB. The Young’s modulus and tensile strength of the BOSB were both about three times those of the WOSB [19]. The changes were the same according to the stiffness analysis. This suggests that the joints’ elastic modulus can be used as a good indicator for the deformation resistance of components. The joint elastic modulus dates of the components fixed with screws were fitted using the exponential function *y = a − bc^x^*, and the fitting results are shown in Table 4. In the table, it can be seen that the fitting degree R^2^ can reach more than 99%, and the fitting effect is very good.

**Figure 8:**
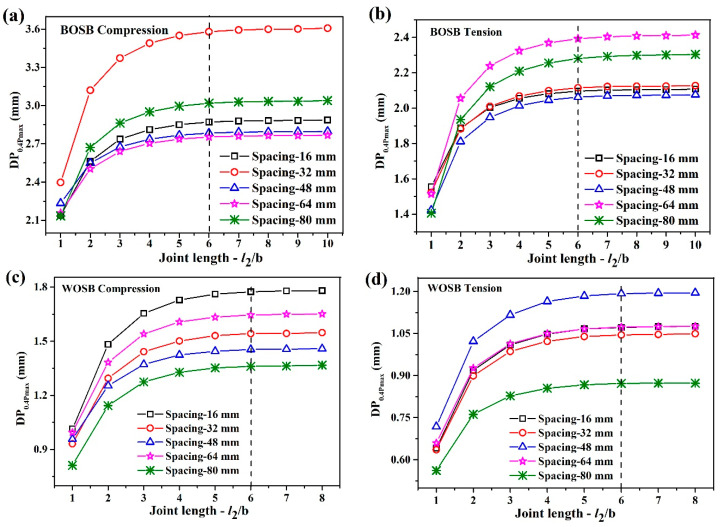
The deformations of the components fixed with screws: (**a**) BOSB—compression; (**b**) BOSB—tension; (**c**) WOSB—compression; (**d**) WOSB—tension.

The simplified model of components was imported into ANSYS Workbench software. The simplified joint model was assigned a joint elastic modulus, while the remaining parts were assigned a material elastic modulus. The load was 0.4 Pmax, and the constraint conditions imposed were identical to those employed in the physical experiments. Figure 8 displays the numerical simulation results illustrating the deflection of the L-shaped components fixed by screws. As the simplified joint’s length increased, the component’s deflection increased. When the length of the simplified joint reached six times the board’s thickness, this deflection stabilized. This result is consistent with the patterns observed in the joint elastic modulus.

**Figure 9:**
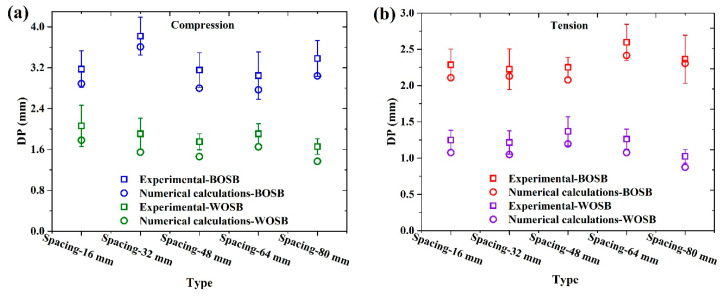
The deformations of screw-fixation components provided by experimental and numerical calculations under (**a**) compression and (**b**) tension.

To verify the accuracy of the FEA, a simplified joint model was used, and its deflection was compared with the experimental results under the same load. The model’s length was six times the board’s thickness. Figure 9 depicts the screw-fixed component’s experimental and numerical deflections under a load of 0.4 Pmax. As shown in the figure, the numerical and experimental results follow the same pattern. Most of the numerical deflection falls within the range of the experimental results, which proves that the FEA is correct. When the components were compressed, the numerical deflections for the BOSB and WOSB were about 5–11% and 13–19% lower than the experimental results. When the components were tensed, the reductions were about 2–8% and 13–15%, respectively. These findings suggest that the numerical and experimental data are more in line with each other for the BOSB components. They also suggest that FEA can simulate BOSB furniture structures more effectively than WOSB ones. Furthermore, it is worth noting that this simplified model method significantly reduces the time requirements for FEA in comparison to using actual models, highlighting its precision and efficiency.

**Figure 10:**
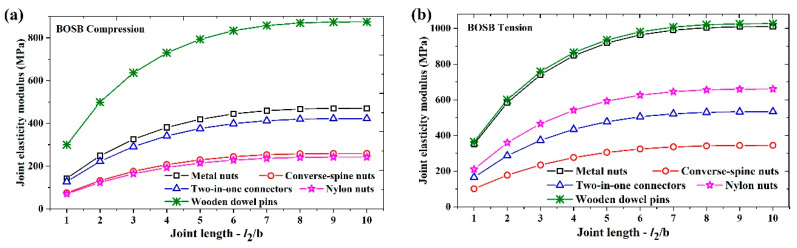

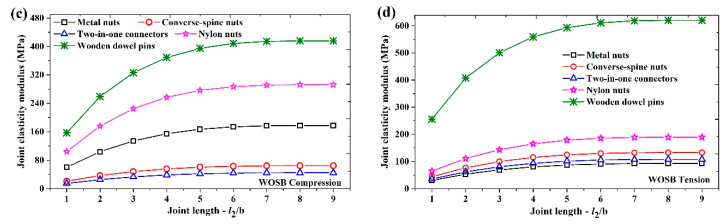
The joint elastic moduli of components fixed with connectors: (**a**) BOSB—compression; (**b**) BOSB—tension; (**c**) WOSB—compression; (**d**) WOSB—tension.

This paper evaluates the applicability of the model simplification method by analyzing L-shaped components with common eccentric connections and wood dowel pins commonly found on the market. Figure 10 presents the joint elastic moduli of a simplified joint fixed with five common connectors. It is evident from the figure that as the length of the simplified joint increased, the joint elastic modulus of the components also increased and gradually stabilized. Figure 10a shows that the BOSB components attached with wooden dowel pins had the highest compression elastic modulus. Those attached with metal nuts and two-in-one connectors trailed behind. The elastic moduli of those attached with converse-spine and nylon nuts were lower. The tensile elastic moduli were higher for the BOSB components attached with wooden dowel pins and metal nuts, whereas they were lower for those attached with converse-spine nuts. Regarding the WOSB components, those fixed with wooden dowel pins had the highest joint elastic modulus, followed by those secured using nylon nuts, while those connected using two-in-one connectors displayed lower values. In addition, the elastic modulus of the components fixed with a common connection exhibited a similar variation trend to stiffness. Table 5 shows the fitting results between the joint elastic modulus and the joint length *l*_2_/b of the components fixed with connectors using the exponential function *y = a − bc^x^*. In the table, it is shown that the fitting coefficients *R*^2^ are all greater than 0.99, indicating that the equation is very well fitted.

**Figure 11:**
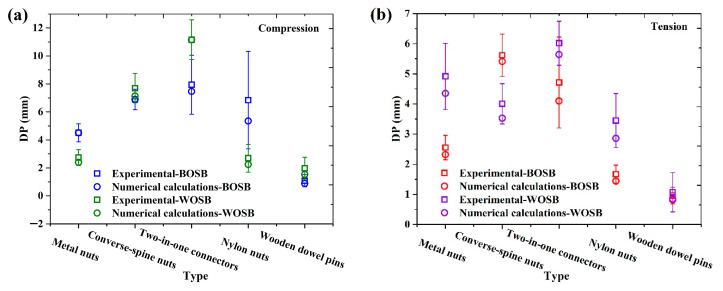
Deformations of connector fixation components provided by experimental and numerical calculations under (**a**) compression and (**b**) tension.

The FEA program imported the simplified model of screw-secured L-shaped components. Then, a load of 0.4 times the maximum load (Pmax) was applied to the model while maintaining the same restrictions as in the experiment. Next, the numerical deflection was compared with the experimental results, as depicted in Figure 11. The figure shows that not only do the FEA results exhibit similar variation trends as the experimental results, but they also show smaller discrepancies in values.

Particularly, the components fastened with nylon nuts and wooden dowel pins showed a larger deflection difference between the numerical and experimental results, whereas those fastened with metal nuts and converse-spine nuts showed a smaller difference. The numerical simulation findings for the BOSB components, when secured by metal nuts and subjected to compressive and tensile stresses, showed decreases of 0.08% and 9%, respectively, compared to the experimental data. For the components secured with converse-spine nuts, the differences were 0.2% and 0.3%, respectively. In terms of the WOSB components fixed by two-in-one connectors, the numerical simulation showed the best accuracy, with differences of about 0.02% and 6% from the experimental results. Taken together, these results indicate that the model simplification method is also applicable to L-type components fixed by common connectors, further demonstrating the effectiveness of the method.

### 3.3. Strength of Joints

**Figure 12:**
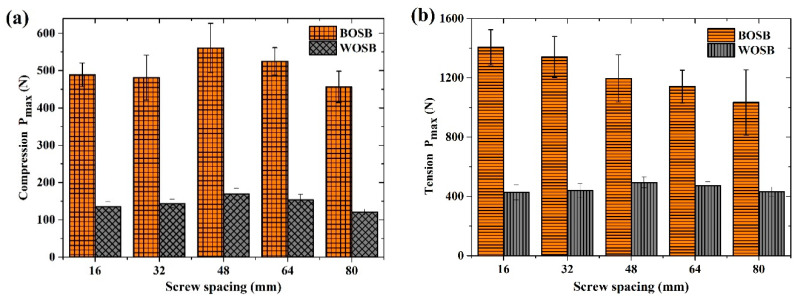
Strength of joints fixed with screws under (**a**) compression and (**b**) tension.

The L-shaped components used in the experiment had uniform dimensions, and the ultimate load represented their strength. Figure 12 shows the strength of the components fixed with differently spaced screws. Figure 12a shows that the compressive strength of the L-shaped component decreased initially and then increased as the screw spacing increased. The maximum load was achieved with a screw spacing of 48 mm, resulting in compressive ultimate loads of 561 N for the BOSB components and 169 N for the WOSB components. Figure 12b presents the ultimate tensile load for the L-shaped components. As the screw spacing increased, the tensile strength of the BOSB components gradually decreased, while that of the WOSB components first increased and then decreased, reaching its maximum at a spacing of 48 mm. This demonstrates that screw spacing has a significant influence on the strength of BOSB components [29]. The trends for these two kinds of board components differed mostly due to their failure mechanisms: BOSB components fail due to bending screws, while WOSB components fail due to board failure [19]. The analysis of variance (Table 6 and Table 7) revealed substantial differences in the maximum loads for the components connected with different screw spacings. The compressive strength of the WOSB components was more influenced by the screw spacing compared to that of the BOSB components. However, in tension, the impact of screw spacing on the WOSB components was comparatively weaker than on the BOSB components. This confirms that the screw spacing directly affects the bending strength.

Compared to the WOSB component, the BOSB component was significantly stronger due to its 3.5-fold higher tensile strength. This is because of the strong dependence between the load of the connections and the density of the boards [32]. Under compression, the ultimate load of the BOSB component was approximately 3.3–3.7 times greater than that of the WOSB component, whereas under tension, it was about 2.4–3.3 times greater. Furthermore, the tensile strength of the BOSB component was 2.4–3.3 times higher than the compressive strength, whereas that of the WOSB component was 3–3.6 times higher. In furniture structural design, it is undesirable to have a significant difference in the component strength under different load conditions. This is because furniture components experience both tension and compression, and they need to have similar stiffness to ensure greater structural reliability [33]. The results indicate that the L-shaped components of BOSB are not only significantly stronger than those of WOSB but also more stable.

**Figure 13:**
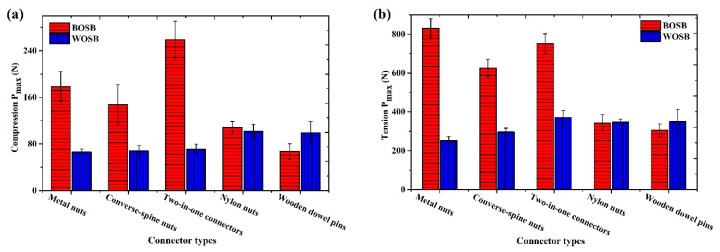
Strength of components fixed with different connectors under (**a**) compression and (**b**) tension.

Figure 13 shows the strength of the L-shaped components with common eccentric connections and wood dowel pin fixings. By comparing the two figures, it is evident that the tensile strength of the components is considerably higher than their compressive strength. Among them, the BOSB component had a tensile strength of 2.9–4.7 times its compressive strength, while that of the WOSB component was 3.4–5.2 times higher. Similar to the components fixed with screws, the strength of the BOSB fixed by common connectors was more stable than that of the WOSB. Unlike the screw-fixed components, there were cases where the strength of the BOSB components was less than that of the WOSB components. For example, under compression, the ultimate load of the WOSB component fixed by the wooden dowel pin was 99 N, which is obviously higher than that of the BOSB component (67 N). Under tension, the ultimate loads for the WOSB component fixed with a nylon nut and wooden dowel pin reached 347 N and 350 N, respectively, which are slightly greater than those of the BOSB component (343 N and 305 N). This indicates that, apart from the material strength, the type and performance of the connector also affect the L-shaped component strength [34], and different material choices with suitable connectors can improve the component’s bearing capacity [14]. The analysis of variance (Table 8 and Table 9) shows that the components fixed by different connectors had significant strength differences. The difference in the type of connectors had a significantly greater effect on the strength of the BOSB components than on that of WOSB components (F (BOSB) > F (WOSB), which suggests that the selection of appropriate connectors is more effective in improving the structural strength of BOSB components.

The compressive strength of L-shaped components attached to different connectors is shown in Figure 13a. The BOSB components attached with a two-in-one connector had the highest compressive strength (259 N), followed by those attached with metal nuts (178 N) and converse-spine nuts (147 N). The compressive strength of those attached with nylon nuts (108 N) and wooden dowel pins (67 N) was lower. The WOSB components fixed with metal nuts, a converse-spine nut, and a two-in-one connector had a lower compressive strength (106 N and 99 N, respectively), whereas those fixed with nylon nuts and wooden dowel pins had higher compressive strength, at 102 N and 99 N, respectively. This further indicates that the different materials are suitable for different connectors. Two-in-one connectors and metal nuts use threads embedded in the board to achieve connection. The BOSB material had a higher density, strength, and hardness than the WOSB; the threads grip the board tightly, so these components have a higher compressive strength [35]. Screwing threaded connectors, such as two-in-one connectors, metal nuts, and converse-spine nuts, into WOSB causes damage and loosens the surrounding material. This weakens the connection between the connectors and the board, thereby reducing the components’ strength [36]. On the other hand, when embedded in WOSB, nylon nuts and wooden dowel pins can create more friction with the material, thereby improving board fixation.

According to Figure 12b, the BOSB component with a metal nut, a two-in-one connection, and a converse-spine nut had substantially greater tensile strength than the one with a nylon nut and a wooden dowel pin. The WOSB component with a nylon nut and wooden dowel pin had somewhat higher tensile strength than the one with a metal nut and a converse-spine nut. The eccentric connection and wooden dowel pin-fixed L-shaped component were weaker than the screw-fixed one. The compressive and tensile strengths of the BOSB components fixed with a 48 mm screw spacing were 3.1 and 1.4 times higher than those of the components fixed with metal nuts. Compared to the nylon nuts, the WOSB components secured with a 48 mm screw spacing were 1.7 and 1.4 times stronger. Compared to two-in-one, three-in-one (with an embedded nut), wood screw, and other threaded connectors, the thread end of self-drilling screws is narrower, the thread spacing is smaller, and the thread has a greater effect on the shear and extrusion deformation of bamboo fiber, so its connection strength is greater than other threaded connectors [14].

### 3.4. Stiffness of Joints

**Figure 14:**
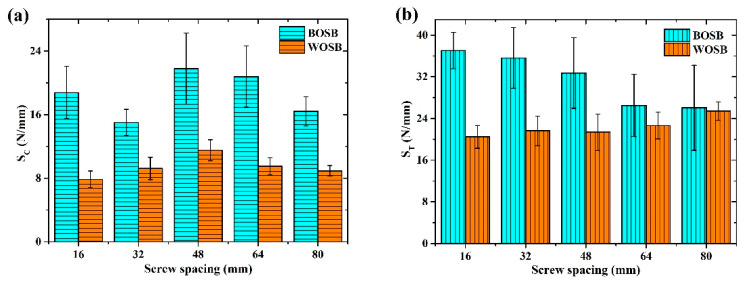
Stiffness of components fixed with screws subjected to (**a**) compression and (**b**) tension.

Figure 13a,b show the stiffness of the L-shaped BOSB and WOSB components fixed by screws with different spacings. Figure 14a shows that the BOSB and WOSB components’ compressive stiffness increased and then decreased with the screw spacing. The compressive stiffness of both the BOSB and WOSB components was at the maximum with a screw spacing of 48 mm, with stiffnesses of 21.8 N/mm and 11.5 N/mm, respectively. Figure 14b shows that the BOSB component’s tensile stiffness decreased as the screw spacing increased, whereas that of the WOSB increased gradually. This shows that the screw spacing affected the components’ stiffness and had a slightly different pattern than that of the strength. Table 10 and Table 11 demonstrate significant differences in the stiffness of the components fixed with varying screw spacings. This proves that screw spacing affects components’ stiffness. Furthermore, screw spacing affects the tensile stiffness more than the compressive stiffness and affects the compressive stiffness of BOSB components more than that of WOSB components, while the effect on the tensile stiffness of BOSB components is slightly less than that of WOSB components.

The tensile stiffness was significantly greater than the compressive stiffness, and the stiffness of the BOSB components was higher than that of the WOSB components. The tensile stiffness of the BOSB components was about 1.3–2.4 times their compressive stiffness, with the smallest difference noted for the component with a screw spacing of 64 mm, followed by 48 mm. Regarding the WOSB components, their tensile stiffness was roughly 1.9–2.8 times greater than their compressive stiffness, with the smallest difference for components with a screw spacing of 48 mm. This shows that BOSB component stiffness is more stable [33]. In addition, the difference in the component stiffness between the two different loads was small compared to that of the component strength.

**Figure 15:**
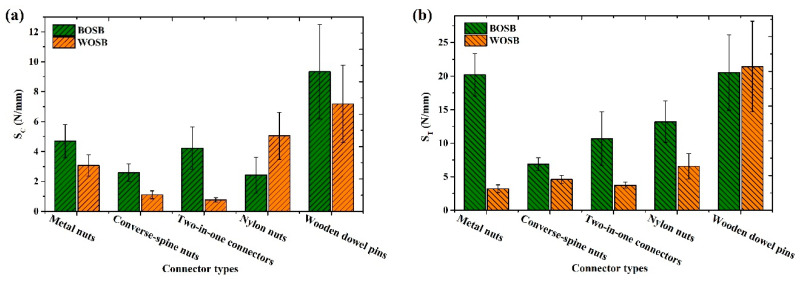
Stiffness of components fixed with connectors subjected to (**a**) compression and (**b**) tension.

Figure 15 shows the stiffness of an L-shaped component fixed with eccentric connections and wooden dowel pins. The comparison revealed that the tensile stiffness of the components was significantly greater than the compressive stiffness. This was the same pattern as the components’ strength. Moreover, the stiffnesses of both the BOSB and WOSB components fixed by wooden dowel pins were at the maximum under two different loads. Figure 15a demonstrates that when compressed, the stiffness of the metal nut-, converse-spine nut-, two-in-one connector-, and wooden dowel pin-fixed BOSB components surpassed that of the WOSB components. BOSB with high hardness is more suitable for thread connections to obtain greater mechanical strength compared to non-threaded connections [35,37]. However, the compressive stiffness of the nylon nut-fixed BOSB components was less than that of the WOSB components. The wooden dowel pin-fixed BOSB component had the greatest compressive stiffness (9.3 N/mm), followed by those fixed with the metal nut (4.7 N/mm) and the two-in-one connector (4.2 N/mm). the nylon nuts-fixed BOSB component had the lowest stiffness (2.4 N/mm). For the WOSB component, the compressive stiffness of the wooden dowel pin fixation was again the highest, followed by that of the nylon nut (5.1 N/mm); that of the two-in-one connector was the lowest (0.8 N/mm). Figure 14b shows that the stiffness of the BOSB components fixed by eccentric connections was much greater than that of the WOSB components. Moreover, for the BOSB components, the tensile stiffness was relatively high for those fixed with metal nuts and wooden dowel pins at 20.2 N/mm and 20.5 N/mm, respectively, followed by those fixed with nylon nuts and two-in-one connectors. The BOSB components fixed with converse-spine nuts had the lowest tensile stiffness. Among the WOSB components, the tensile stiffness of the wooden dowel pin-fixed component was the highest (21.4 N/mm), followed by that with the nylon nut; that of the metal nut was the lowest (3.2 N/mm). The analysis of variance (ANOVA) in Table 12 and Table 13 shows that the type of connector affects the component stiffness, with a greater effect on the stiffness of WOSB components than on that of BOSB components.

In summary, the wooden dowel pin-fixed BOSB component exhibited the highest stiffness; however, its ultimate load was comparatively lower. Therefore, wooden dowel pin fixation is not an optimal choice for BOSB components. Metal nut fixation is a more suitable option for BOSB components, with a stiffness slightly lower than that of wooden dowel pin fixation, and compressive and tensile ultimate loads approximately 2.7 times higher. For WOSB components, it is more suitable to fix them with wooden dowel pins, which not only have higher strength but also better stiffness.

## 4. Conclusions

The following deductions were made:

(1) The theoretical deflection calculation method for cantilever beams can be applied to simplify models of L-type components and establish a formula for the joint elastic modulus, which is instrumental in assessing the deformation resistance of L-shaped components. Comparing the numerical results with the experimental results confirmed the model simplification method’s accuracy and validity and its applicability in the analysis of components fixed by multiple connectors. Notably, it was observed that the joint elastic modulus of the components increased as the simplified joint length increased, stabilizing when *l*_2_/*b* ≥ 6 times higher.
(2) After simplifying the model, the numerical results of the L-shaped component’s deflection align with the experimental findings. Specifically, the numerical deflections of the screw-fixed BOSB components under compression and tension were lower by about 5–11% and 2–8%, respectively, than the experimental results, whose lower values were smaller than those of the WOSB components. Moreover, under compressive and tensile loads, the numerical deflections of the BOSB components fixed by metal nuts were lower by 0.08% and 9% than the experimental results, respectively, while those fixed by converse-spine nuts exhibited differences of 0.2% and 0.3%.
(3) The screw spacing had a significant effect on the L-shaped components’ strength and stiffness. As the screw spacing increased, both the BOSB and WOSB components’ compressive ultimate loads and stiffnesses increased and then decreased. However, the ultimate tensile load and stiffness of the BOSB components gradually decreased over time. Moreover, both the BOSB and WOSB components had better strength and stiffness and were more stable when the screw spacing was 48 mm. In addition, the ultimate load of the BOSB component was about 2.4–3.7 times that of the WOSB component, while the difference in stiffness between the two components was small.
(4) The connector type and performance also affected the strength and stiffness of the L-shaped components, especially for the BOSB components. Choosing suitable connectors was more helpful in improving the components’ bearing capacity. For example, two-in-one connectors and metal nuts secured the BOSB components, utilizing threads embedded in the board for superior strength and stiffness, while nylon nuts and wooden dowel pins were more suitable for securing the WOSB components. In addition, the strength and stiffness of the components fixed with common eccentric connections and wood dowel pins were lower than those secured with screws. Moreover, the bearing capacity of the BOSB components was more consistent than that of the WOSB components.

The use of single specimen dimensions and the fact that the model simplification was not applied to finished furniture for validation are the limitations of this study. Therefore, these need to be further studied. In the future, we will continue to study the application of FEA in the structural performance evaluation of furniture to improve its accuracy, efficiency, and applicability.

## Figures and Tables

**Table 1:** Physical and mechanical properties of boards.

Material	Thickness/Diameter (mm)	Density (kg/m^3^)	Young’s Modulus (Mpa)	Tensile Strength (Mpa)	Poisson’s Ratio	Moisture Content (%)
BOSB	15	806.61	6770.86	32.09	0.33	7.84
WOSB	18	569.2	2096.15	9.1	0.32	6.05

**Table 2:** Grade specifications of connectors.

Name	Manufacturer	Material	Length/mm	Outside Diameter/mm	Inside Diameter/mm
Screw	Fastening Star Hardware	Stainless steel	40	3.97	2.76
Metal nut	Guangzhou Haidi Bosi Hardware	Stainless steel	7	10	6.78
Nylon nut	Guangzhou Haidi Bosi Hardware	Polyethylene	10	11	6
Converse-spine nut	Jingong Hardware	Stainless steel	10	8.5	5.3
Two-in-one connector	Guangdong Xiangzhen Hardware	Stainless steel	44	11	7
Wooden dowel pin	Guangzhou Haidi Bosi Hardware	Poplar	40	8	

**Table 3:** Installation dimensions of connectors.

Name	Depth/mm	Aperture Ratio
Screw	10	0.85
Metal nut	7	0.95
Nylon nut	10	0.87
Two-in-one connector	10	0.51
Converse-spine nut	10	0.95
Wooden dowel pin	10	0.96

**Table 4:** The fitting equations between the joint elastic modulus and the joint length *l*_2_/*b* of the components fixed with screws.

Type		Parameters			R^2^
		a	b	c	
BOSB—Compression	Spacing—16 mm	1887.78	1729.30	0.60	0.9998
	Spacing—32 mm	1509.06	1479.58	0.62	0.9995
	Spacing—48 mm	2219.40	1829.49	0.57	0.9997
	Spacing—64 mm	2105.88	1816.98	0.58	0.9998
	Spacing—80 mm	1682.88	1611.50	0.61	0.9997
BOSB—Tension	Spacing—16 mm	1894.39	1751.32	0.60	0.9998
	Spacing—32 mm	1792.46	1678.01	0.60	0.9998
	Spacing—48 mm	1639.79	1567.67	0.61	0.9997
	Spacing—64 mm	1362.28	1354.53	0.63	0.9994
	Spacing—80 mm	1289.93	1281.04	0.63	0.9994
WOSB—Compression	Spacing—16 mm	455.16	513.03	0.59	0.9987
	Spacing—32 mm	554.82	629.12	0.57	0.9991
	Spacing—48 mm	688.64	789.52	0.56	0.9994
	Spacing—64 mm	556.62	631.23	0.57	0.9991
	Spacing—80 mm	527.03	596.46	0.58	0.9990
WOSB—Tension	Spacing—16 mm	603.88	631.23	0.59	0.9984
	Spacing—32 mm	637.31	662.18	0.58	0.9985
	Spacing—48 mm	629.58	654.10	0.58	0.9985
	Spacing—64 mm	667.04	690.15	0.58	0.9986
	Spacing—80 mm	746.71	762.72	0.58	0.9988

**Table 5:** The fitting equations between the joint elastic modulus and the joint length *l*_2_/*b* of the components fixed with connectors.

Type		Parameters			R^2^
		a	b	c	
BOSB—Compression	Metal nuts	487.21	517.13	0.67	0.9980
	Converse-spine nuts	269.85	289.44	0.68	0.9976
	Two-in-one connectors	437.71	465.25	0.67	0.9980
	Nylon nuts	251.96	269.93	0.68	0.9976
	Wooden dowel pins	897.00	923.38	0.65	0.9989
BOSB—Tension	Metal nuts	1034.37	1060.84	0.65	0.9990
	Converse-spine nuts	357.73	382.39	0.68	0.9977
	Two-in-one connectors	551.23	581.35	0.67	0.9982
	Nylon nuts	680.92	714.84	0.66	0.9984
	Wooden dowel pins	1050.10	1069.73	0.64	0.9991
WOSB—Compression	Metal nuts	182.88	200.51	0.62	0.9969
	Converse-spine nuts	66.53	73.65	0.63	0.9965
	Two-in-one connectors	46.14	51.17	0.63	0.9964
	Nylon nuts	300.14	324.81	0.61	0.9974
	Wooden dowel pins	425.23	452.30	0.60	0.9980
WOSB—Tension	Metal nuts	95.90	105.94	0.62	0.9966
	Converse-spine nuts	136.94	150.80	0.62	0.9967
	Two-in-one connectors	111.33	122.86	0.62	0.9966
	Nylon nuts	195.13	213.59	0.62	0.9970
	Wooden dowel pins	631.19	649.56	0.58	0.9986

**Table 6:** ANOVA results of compression strength of joints fixed with screws.

Material	Source of Variation	Sum of Squares	df	Mean Square	F	Level of Significance
BOSB	Between groups	53,284.904	4	13,321.226	5.521	0.001
	Within groups	84,447.355	35	2412.782		
	Total	137,732.259	39			
WOSB	Between groups	10,683.858	4	2670.964	16.139	0.000
	Within groups	5792.437	35	165.498		
	Total	16,476.295	39			

**Table 7:** ANOVA results of tension stiffness of joints fixed with screws.

Material	Source of Variation	Sum of Squares	df	Mean Square	F	Level of Significance
BOSB	Between groups	723,347.638	4	180,836.909	7.550	0.000
	Within groups	838,325.715	35	23,952.163		
	Total	1,561,673.353	39			
WOSB	Between groups	26,717.239	4	6679.310	4.240	0.007
	Within groups	55,133.698	35	1575.249		
	Total	81,850.937	39			

**Table 8:** ANOVA results of compression strength of components fixed with different connectors.

Material	Source of Variation	Sum of Squares	df	Mean Square	F	Level of Significance
BOSB	Between groups	169,809.476	4	42,452.369	69.223	0.000
	Within groups	21,464.530	35	613.272		
	Total	191,274.006	39			
WOSB	Between groups	9935.890	4	2483.973	17.576	0.000
	Within groups	4946.427	35	141.326		
	Total	14,882.318	39			

**Table 9:** ANOVA results of tension stiffness of components fixed with different connectors.

Material	Source of Variation	Sum of Squares	df	Mean Square	F	Level of Significance
BOSB	Between groups	1,805,732.642	4	451,433.161	231.521	0.000
	Within groups	68,245.001	35	1949.857		
	Total	1,873,977.643	39			
WOSB	Between groups	74,594.608	4	18,648.652	14.299	0.000
	Within groups	45,648.022	35	1304.229		
	Total	120,242.630	39			

**Table 10:** ANOVA results of compressive stiffness of components fixed with screws.

Material	Source of Variation	Sum of Squares	df	Mean Square	F	Level of Significance
BOSB	Between groups	261.207	4	65.302	6.292	0.001
	Within groups	363.262	35	10.379		
	Total	624.469	39			
WOSB	Between groups	57.479	4	14.370	11.141	0.000
	Within groups	45.143	35	1.290		
	Total	102.622	39			

**Table 11:** ANOVA results of tension stiffness of components fixed with screws.

Material	Source of Variation	Sum of Squares	df	Mean Square	F	Level of Significance
BOSB	Between groups	829.930	4	207.483	5.333	0.002
	Within groups	1361.715	35	38.906		
	Total	2191.646	39			
WOSB	Between groups	115.079	4	28.770	4.140	0.008
	Within groups	243.237	35	6.950		
	Total	358.316	39			

**Table 12:** ANOVA results of compressive stiffness of joints fixed with connectors.

Material	Source of Variation	Sum of Squares	df	Mean Square	F	Level of Significance
BOSB	Between groups	250.687	4	62.672	20.997	0.000
	Within groups	104.466	35	2.985		
	Total	355.153	39			
WOSB	Between groups	234.307	4	58.577	29.930	0.000
	Within groups	68.499	35	1.957		
	Total	302.806	39			

**Table 13:** ANOVA results of tension stiffness of joints fixed with connectors.

Material	Source of Variation	Sum of Squares	df	Mean Square	F	Level of Significance
BOSB	Between groups	1143.599	4	285.900	20.910	0.000
	Within groups	478.543	35	13.673		
	Total	1622.142	39			
WOSB	Between groups	1883.119	4	470.780	47.045	0.000
	Within groups	350.242	35	10.007		
	Total	2233.361	39			

## Data Availability

The raw/processed data required to reproduce these findings cannot be shared at this time as the data also form part of an ongoing study.
